# Chemical Profile and Potential Applications of *Sclerocarya birrea* (A.Rich.) Hochst. subsp. caffra (Sond.) Kokwaro Kernel Oils: Analysis of Volatile Compounds and Fatty Acids

**DOI:** 10.3390/molecules29163815

**Published:** 2024-08-11

**Authors:** Callistus Bvenura, Learnmore Kambizi

**Affiliations:** Department of Horticulture, Faculty of Applied Sciences, Cape Peninsula University of Technology, Bellville 7535, South Africa; kambizil@cput.ac.za

**Keywords:** cosmetics, FAMEs, fatty acids, marula kernel oil, *Sclerocarya birrea*, volatile compounds

## Abstract

*Sclerocarya birrea* kernel volatile compounds and fatty acid methyl esters (FAMEs) from the Bubi district in Matabeleland North province of Zimbabwe were characterised by GC–MS. The volatile compounds of the oil include 65 different compounds from 24 distinct classes, dominated by 13 alcohols and 14 aldehydes (42%). Other classes include carboxylic acids, phenols, sesquiterpenes, lactones, pyridines, saturated fatty acids, ketones, and various hydrocarbons. The kernel oils revealed essential fatty acids such as polyunsaturated (α-linolenic and linoleic acids) and monounsaturated fatty acids (palmitic, palmitoleic, and oleic acids). Notably, oleic acid is the predominant fatty acid at 521.61 mg/g, constituting approximately 73% of the total fatty acids. Linoleic acid makes up 8%, and saturated fatty acids make up about 7%, including significant amounts of stearic (42.45 mg/g) and arachidic (3.46 mg/g) acids. These results validate the use of marula oils in food, pharmaceutical, and health industries, as well as in the multibillion USD cosmetics industry. Therefore, the potential applications of *S. berria* kernel oils are extensive, necessitating further research and exploration to fully unlock their capabilities.

## 1. Introduction

*Sclerocarya birrea* (A.Rich.) Hochst. subsp. caffra (Sond.) Kokwaro, better known as marula, is an important multipurpose African savanna tree belonging to the family Anacardiaceae and widely distributed in sub-Saharan Africa. The entire tree is useful for food, forage/feed, medicine, and furniture; for example, the fruit is eaten either fresh or made into a jelly, and the pulp is fermented into a culturally important traditional alcoholic beverage known as ‘*ubuganu*’ in Eswatini or ‘mukumbi’ in Zimbabwe and some parts of South Africa. In fact, a festival known as ‘*Buganu*’ is annually held in Eswatini from mid-February to May to celebrate the marula harvest, marking the significance of this fruit. But a more exquisite ‘marula cream liqueur’ has been produced and is sold internationally. The leaves are browsed by game and the bark stripped by elephants for food. The bark and roots are widely used as an antihistamine and possesses anti-diarrhoea properties, among others, while the wood is used for making household utensils [1]. The kernels of the stones are edible, and oils are extracted for culinary, cosmetic, and medicinal purposes [2].

Marula and its products have acquired a significant value especially in the South African market, leading to the formation of a ‘marula sector’ concerned with the exploitation of marula products such as beverages and oils. The global essential oil market is currently valued at USD 12.47 billion and is expected to rise to USD 27.82 billion by 2032 [3]. Marula oils are an essential component of this market: therefore, their constituent volatile compounds and expressed oils need to be investigated. Essential oils are widely used as flavourants, fragrances in shampoos and lotions, for skin and hair rejuvenation, in soap, candles, as perfumes, in sterilising/sanitising liquids, and in medical or conventional aromatherapy, among other uses [4]. But the lack of scientific validation of aesthetic aromas, volatile compounds, and extracts for human health is a current hotpot of criticism of natural or organic-based therapies. Despite this, substantial scientific evidence exists demonstrating modest to significant biological effects from aromatic plant-based cosmetics, nutraceuticals, and therapies. Therefore, marula oils that form part of the multibillion USD industry need to be surfaced and validated to enhance their value for industrial applications and product development.

Techniques such as a Fourier-transform infrared spectroscopy (FTIR) and gas chromatography–mass spectrometry (GC–MS) are used to analyse volatile substances of kernel oil. GC–MS separates and identifies the complex volatile compounds and provides detailed qualitative and quantitative data. In so doing, the major volatile compounds including esters, alcohols, and terpenes are profiled. Terpenes contribute significantly to the oil’s fragrance, alcohols enhance scent and stability, and esters add pleasant fruity flavours, defining the oil’s aromatic profile [5,6].

To analyse fatty acid profiles, gas chromatography with flame ionization detection (GC-FID) or gas chromatography–mass spectrometry (GC–MS) are used. These techniques separate, identify, and quantify fatty acids in their methyl ester forms (FAMEs), consequently providing detailed compositions of saturated, monounsaturated, and polyunsaturated fatty acids. These fatty acids are critical in understanding the nutritional and industrial value of the oil. For example, saturated fatty acids, while contributing to the stability of the oil [7], must be balanced with unsaturated fatty acids, which are known for their health benefits and desirable physical properties [8,9].

Therefore, the present work was conducted to profile the *Sclerocarya birrea* kernel volatile constituents of kernel oil and FAMEs from the species growing wild in the Mbembesi area of Matabeleland South province of Zimbabwe.

## 2. Results

### 2.1. Volatile Constituents of Kernel Oil

The volatile constituents of kernel oil are composed of a total of 65 different compounds consisting of 24 distinct classes including 13 alcohols, 14 aldehydes, 5 carboxylic acids, 4 phenols, 4 sesquiterpene, 4 lactones, 2 pyridines, 2 saturated fatty acids, 2 ketones, and an alkylbenzene, alkyne, cyclic ketone, cycloalkene, diene, diketone, furan, glycol ether, guaiacol, halogenated benzene, medium-chain fatty acid, monoterpene, phenolic compound, and a terpene, as well as a vinyl aromatic hydrocarbon. Alcohols and aldehydes dominated (42%) the compounds (Table 1).

### 2.2. Fatty Acids Methyl Esters (FAMEs)

Our analysis showed that the kernel oils are rich in essential fatty acids including polyunsaturated fatty acids (αlinolenic and linoleic acids) and monounsaturated fatty acids, which included palmitic, palmitoleic, and oleic acids (Table 2). However, about 11 non-essential saturated fatty acids were also detected but in very small quantities, except for (mg/g) stearic (42.45), arachidic (3.46), and cis-11-Eicosenoic (1.37) acid. In contrast, (mg/g) oleic (521.61), palmitic (81.072), and linoleic (55.54) acid were very abundant in the oil. Oleic acid is thus the predominant fatty acid in marula oil, comprising approximately 73% of the total fatty acids, while linoleic acid constitutes about 8%. Saturated fatty acids constitute about 7% of the total fatty acid content in marula oil, as seen in this study.

## 3. Discussion

### 3.1. Volatile Constituents of Kernel Oil

According to the International Standards Organisation, a single organic compound like limonene is not an essential oil but rather a volatile organic compound commonly found in essential oils [4]. This distinction, termed ‘essential oil component’ by [10], is crucial. Essential oils are thus mixtures of volatile organic compounds obtained through distillation from aromatic species, including bryophytes like liverworts and higher plants.

Studies on volatile constituents or compounds of *Sclerocarya birrea* kernels are scarce; however, the identification and quantification of volatile compounds not only defines the aroma profile of marula seed oil but also helps in understanding its potential sensory attributes as well as its potential in the pharmaceutical industry, particularly the multibillion USD cosmetics industry. Comparing with other studies, the work of [11] previously profiled 49 compounds, of which 96% were sesquiterpenes, albeit from the leaves. Only four sesquiterpenes were reported in the present study while alcohols and aldehydes dominated the profiles.

The aroma profile, characterised by a balance of sweet, floral, and fruity notes, can be attributed largely to alcohols, aldehydes, ketones, and terpenes reported in the present work, and more specifically, constituents such as1-Hexanol, Trans-2-hexenal, 2-Nonanone, and limonene [4,12]. These compounds were analysed for their concentration and impact on the overall fragrance, providing valuable information for applications in cosmetics and aromatherapy.

In the present study, we presented some of the most common antimicrobial preservatives in the industry, including numerous individual alcohols, phenols, ketones, terpenes, and aldehydes, among others [13].

The role of alcohols in industry and pharmacology are diverse, for example, 1-butanol, which we report in the present study, is extensively used as an artificial flavour additive in the food and beverage industries, as well as a crucial solvent, chemical intermediate, and extractant in cosmetics and pharmaceuticals [14]. Furthermore, this compound is employed in the synthesis of butyl acrylate and methacrylate esters for latex coatings, enamels, and lacquers, as well as in producing butyl glycol ether, butyl acetate, and plasticisers. Additionally, it serves as a diluent in brake fluid formulation and as a solvent in the production of hormones, vitamins, and antibiotics [14]. As such, marula volatile compounds are a promising source of fuel, medicine, and food components.

Aldehydes are highly reactive and toxic chemicals used in industry and derived from alcohol through dehydrogenation [15]. None-the-less, although some aldehydes such as decanal and octanal are used as perfume components, they have been shown to exhibit inhibitory and bactericidal effects on *Escherichia coli*, *Staphylococcus aureus*, *Saccharomyces cerivisiae*, and *Aspergillus niger* [16]. This further establishes marula kernel oils as valuable in the development of new organic medicinal interventions in the face of ever-increasing drug-resistant pathogens.

Terpenes are the primary components of essential oils, characterised by carbon backbones derived from 2-methylbuta-1,3-diene (isoprene units), whose number determines the structural diversity into hemiterpenes (C5), monoterpenes (C10), sesquiterpenes (C15), diterpenes (C20), triterpenes (C30), and tetraterpenes (C40) [17]. These compounds have significant roles in treating various diseases, demonstrated in numerous in vitro and in vivo studies for their anticancer, antimicrobial, anti-inflammatory, antioxidant, antiallergic, neuroprotective, antiaggregatory, anticoagulant, sedative, and analgesic properties [18]. The present study surfaced the presence of monoterpenes. In fact, we report the presence of limonene, one of the main essential-oil components of citrus fruits with wide applications in the food industry as a flavouring agent due to its transparency and characteristic flavour [19]. Therefore, the exploitation of these oils could be of potential benefit to pharmacology and the food industry.

Besides the role of ketones in characterising the aroma of oils as well as its role in the cosmetics industry as perfume components, some studies found that 2-nonanone inhibits the refolding process of heat-inactivated bacterial luciferases, which rely on the DnaKJE-ClpB bichaperone system [20]. These authors found that the inhibitory effect is most pronounced in *E. coli* cells lacking the small chaperone IbpB. These studies show the inhibitory effects of ketones on bacterial survival, i.e., antibacterial properties.

The potential sensory and pharmacological attributes of marula seed oil, influenced by its volatile composition, highlight its utility in creating appealing and effective aromatic products and new potent organic drugs used against drug-resistant pathogens.

### 3.2. Fatty Acids Methyl Esters (FAMEs)

Our analysis identified oleic acid as the predominant fatty acid in marula oil, comprising approximately 73% of the total fatty acids. This is consistent with findings in ‘virgin’ olive oil, which is also high in oleic acid [21]. The high concentration of linoleic acid (about 8%) suggests that marula oil may also be beneficial for skin health, aligning with its traditional use in skincare.

The present results are comparable with other reports. For example, the study of [22] reported similar FAMEs but in higher quantities. The work of [23] reported eleven FAMEs in comparison to the 16 that we reported. Some authors have reported different fatty acids from the current report except for palmitoleic acid [24]. This is probably because the authors’ report is on the pulp and skin of the fruit. However, the report of [25] revealed about 30 fatty acids in the kernels, although their constituents were generally quantitatively smaller than that of our report.

Fatty acids are long-chain hydrocarbons with a methyl group at one end and a carboxyl group at the other [26,27]. They are classified into four categories: saturated, monounsaturated, polyunsaturated, and trans fats. Over 20 types of fatty acids are found in foods sourced from fruits, vegetable oils, seeds, nuts, animal fats, and fish oils [26].

In *Sclerocarya birrea* kernel oil, α- linolenic and linoleic acid (polyunsaturated omega-3 and 6 fatty acids, respectively) and oleic acid (a monounsaturated omega-9 fatty acid) were identified. Essential fatty acids like omega 3 and 6 are crucial for cellular functions and must be obtained through diet, as the body cannot synthesise them [26]. In addition, these fatty acids have significant implications for health, offering benefits such as anti-inflammatory properties and cardiovascular health support. However, saturated fats have been associated with an increase in cardiovascular related risks [28].

In fact, the WHO [29] strongly recommends that adults and children reduce saturated fatty acid intake to 10% of total energy intake. It also strongly advises replacing saturated fats with polyunsaturated fats and conditionally recommends using monounsaturated fats from plant sources or carbohydrates rich in natural dietary fibre such as whole grains, vegetables, fruits, and pulses. The present study shows that *S. berria* kernel oils are rich in both polyunsaturated and monounsaturated fats. The saturated fats found in these kernels are significantly low and should not pose a concern for health.

Noncommunicable diseases (NCDs) are the leading cause of death globally, accounting for 41 million of 55 million deaths in 2019 [30]. Nearly half of these deaths were premature, occurring in individuals under 70, predominantly in low- and middle-income countries. Cardiovascular diseases (CVDs) led NCD mortality in 2019 caused over 18 million deaths [31]. Key modifiable risk factors for CVDs include unhealthy diets, physical inactivity, tobacco use, and harmful alcohol consumption. High intake of dietary saturated fatty acids (SFA) and trans-fatty acids (TFA) significantly increases CVD risk [29]. SFAs, found mainly in animal products and some plant oils, contain only single carbon–carbon bonds. TFAs, with at least one trans double bond, can be industrially produced or naturally found in ruminant animal products. Reducing SFA intake, particularly when replaced by polyunsaturated fatty acids or whole grain carbohydrates, lowers coronary heart disease risk by improving blood lipid profiles, reducing total and LDL cholesterol levels [32]. This further underscores the potential dietary role of *S. berria* and its capacity to lower NCD.

The high oleic acid content enhances the nutritional value of marula oil, making it comparable to other healthy oils like olive oil. The stability of oleic acid and other fatty acids in these oils at high temperatures also makes them highly suitable for culinary use [33]. Additionally, the significant presence of polyunsaturated fatty acids underscores their potential in the cosmetic industry for formulating skin hydration products due to their excellent moisturising properties. More specifically for skin care, oils rich in linoleic and α-linolenic acid are particularly beneficial as they help reduce the occurrence of eczemas and atopic dermatitis [34]. According to these authors, these essential fatty acids integrate into cell membranes, repair the damaged lipid barrier of the epidermis, and minimise water loss.

The comprehensive fatty acid profile obtained through FAME analysis thus underscores the multifunctional applications of marula seed oil in both health and industry.

## 4. Materials and Methods

### 4.1. Sampling

Dry *Sclerocarya birrea* stones were gathered under trees in Village 1, Clonmore area, Bubi rural district council in the Matabeleland North province of Zimbabwe in the month of April 2023, with samples collected from at least 20 different trees. The stones were then taken to the laboratory at the Cape Peninsula University of Technology, Horticultural Sciences Department, and crushed using a hammer to remove the kernels. The kernel oils were then expressed using a Farmet oil press (Jirinková 276, Ceská Skalice, Czech Republic—Type: UNO (2014); serial number: 0684) and stored in the refrigerator at 4 °C until further use.

### 4.2. Volatile Compounds of Kernel Oil

#### 4.2.1. Sample Preparation

About 5 mL of 20% sodium chloride (NaCl) solution was added to the samples (1 g) and vortexed. The samples were loaded onto the sample tray and 1 µL was injected in split-less mode onto the GC–MS instrument and analysed by SPME-GC–MS, using a 50/30 µm DVB/CAR/PDMS—grey fiber. The SPME conditions were as follows: incubation time of 10 min, incubation temperature of 60 °C, and extraction time of 10 min.

#### 4.2.2. Chromatographic Separation

Separation was performed on a gas chromatograph (6890N, Agilent technologies network) coupled to an Agilent technologies inert XL EI/CI Mass Selective Detector (MSD) (5975B, Agilent technologies Inc., Palo Alto, CA, USA). The GC–MS system was coupled to a CTC Analytics PAL autosampler. Separation of the volatile compounds was performed on a ZB-Wax (30 m, 0.25 mm ID, 0.25 µm film thickness) capillary column (Phenomenex, Torrance, CA, USA). Helium was used as the carrier gas at a flow rate of 1.0 mL/min. The injector temperature was maintained at 240 °C. Split-less injection was performed. The oven temperature was programmed as follows: 40 °C for 6 min and ramped to 260 °C at a rate of 8 °C/min and held for 3 min. The MSD was operated in a full scan mode and the source and quad temperatures were maintained at 230 °C and 150 °C, respectively. The transfer line temperature was maintained at 250 °C. The mass spectrometer was operated under electron impact (EI) mode at ionization energy of 70 eV, scanning from 30 to 700 *m*/*z*.

#### 4.2.3. Identification of Compounds

Compound identification relied on mass spectral data of the samples, which were compared with standard NIST and Wiley Library references, along with retention indices. The relative content (%) of each volatile compound was determined by dividing the peak area of each component by the total peak area of all identified compounds.

### 4.3. Fatty Acids Methyl Esters (FAMEs)

#### 4.3.1. Sample Preparation

About 2 mL of a chloroform to methanol (2:1) mixture was added to approximately 25 mg of the sample. The mixture was vortexed and sonicated at room temperature for 30 min. Following this, 3 mL of a 20% NaCl solution was added to the samples and vortexed. The samples were then centrifuged at 4000 rpm for 2 min. Subsequently, 200 µL of the chloroform layer (bottom layer) was dried completely using a gentle stream of nitrogen. It was then reconstituted and vortexed with 170 µL of methyl tert-butyl ether (MTBE) and 30 µL of trimethylsulfonium hydroxide (TMSH) as the reconstituted solvent/solution mixture. Finally, 1 µL of the derivatised sample was injected onto the GC-FID with a split ratio of 5:1.

#### 4.3.2. Chromatographic Separation

Separation was performed on a gas chromatograph (6890N, Agilent Technologies) coupled to flame ionization detector (FID) at 250 °C. Separation of the FAMEs was performed on a polar RT-2560 (100 m, 0.25 mm ID, 0.20 µm film thickness) (Restek, Bellefonte, PA, USA) capillary column. Hydrogen was used as the carrier gas at a flow rate of 1.2 mL/min. The injector temperature was maintained at 240 °C. About 1 µL of the sample was injected in a 5:1 split ratio. The oven temperature was programmed as follows: 100 °C for 4 min, ramped to 240 °C at a rate of 3 °C/min for 10 min.

Internal standard calibration was performed using C19:0 as the internal standard. The FAMEs were identified using the 37 SUPLECO FAME standards—Merck Life Sciences.

## 5. Conclusions

In conclusion, *S. berria* kernel oil, with its unique volatile composition, offers significant sensory and pharmacological benefits, making it suitable for aromatic products and potent organic drugs against drug-resistant pathogens. The FAME analysis of marula oil reveals a rich fatty-acid profile, particularly high in oleic and linoleic acids, suggesting its potential in the food, health, and cosmetic industries. Thus, the implications on applications of marula kernel oils are vast, consequently warranting further research and exploration to fully realise its potential.

## Figures and Tables

**Table 1:** *Sclerocarya birrea* volatile compounds of kernel oil.

	Tentative Identification	Class	* Structure	Retention Time (mins)	Area %
1	1-Butanol	Alcohols	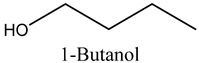	6.08	1.04
2	1-Hexanol	Alcohols	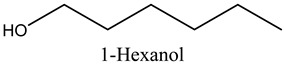	11.53	3.26
3	1-Nonanol	Alcohols	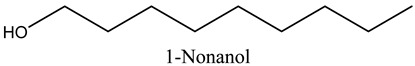	16.69	1.18
4	1-Octen-3-ol	Alcohols	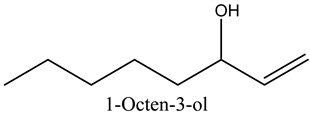	13.38	2.49
5	1-Octen-3-one	Alcohols	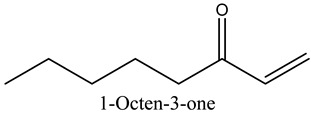	10.28	0.21
6	1-Pentanol	Alcohols	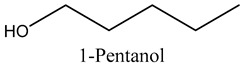	9.25	7.51
7	Heptanol	Alcohols	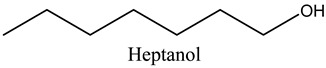	13.44	3.31
8	Octanol	Alcohols	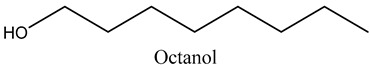	15.14	6.41
9	Phenethyl alcohol	Alcohols	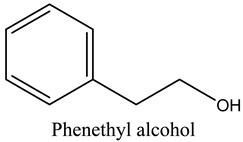	20.09	0.52
10	Trans-2-Octenol	Alcohols	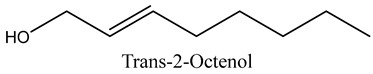	16.03	0.57
11	Decanal	Aldehyde	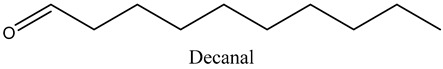	14.11	0.21
12	Heptanal	Aldehydes	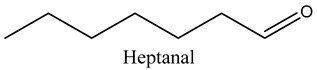	6.95	3.99
13	Nonanal	Aldehydes	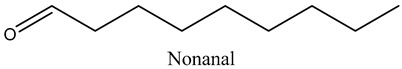	12.21	10.25
14	Trans, cis-2,4-Decadienal	Aldehydes	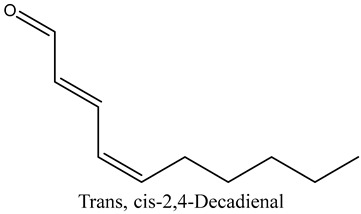	18.15	0.50
15	Trans, trans-2,4 heptadienal	Aldehydes	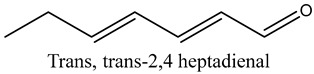	14.00	0.66
16	Trans, trans-2,4-decadienal	Aldehydes	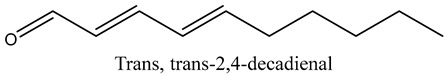	18.74	1.82
17	Trans-2-Decenal	Aldehydes	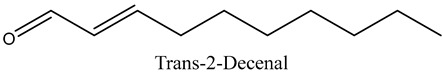	16.37	3.05
18	Trans-2-heptenal	Aldehydes	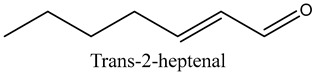	10.71	6.20
19	Trans-2-hexenal	Aldehydes	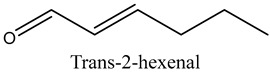	8.02	0.86
20	Trans-2-Nonenal	Aldehydes	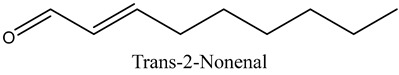	14.68	0.98
21	Trans-2-trans-4-nonadienal	Aldehydes	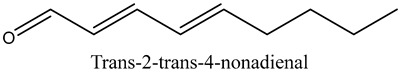	17.22	0.58
22	2-undecenal	Aldehydes	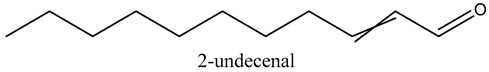	17.94	1.27
23	Octanal	Aldehydes	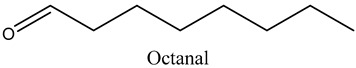	9.95	4.82
24	n-Butylbenzene	Alkylbenzenes	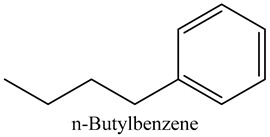	10.36	0.55
25	3-Decyne	Alkynes	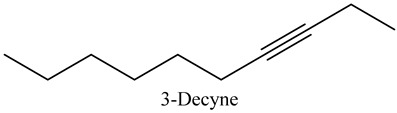	14.16	0.41
26	Benzyl alcohol	Aromatic alcohol	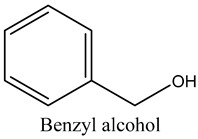	19.65	0.32
27	Benzaldehyde	Aromatic aldehydes	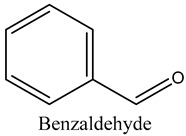	14.46	0.82
28	Isoamyl alcohol	Branched-chain alcohols	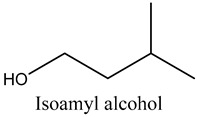	8.07	0.65
29	Butanoic acid	Carboxylic acids	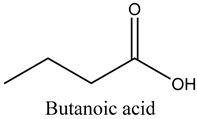	16.22	0.09
30	Octanoic acid	Carboxylic acids	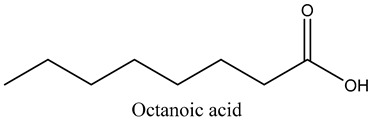	21.95	2.93
31	Valeric acid	Carboxylic acids	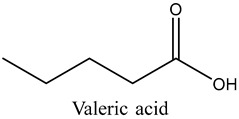	17.79	1.12
32	Isovaleric acid	carboxylic acids	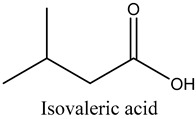	16.82	0.88
33	Nonanoic acid	Carboxylic acids	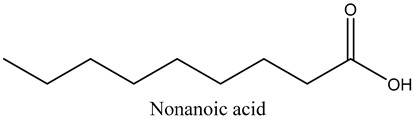	23.20	1.29
34	4-Isopropyl-1,3-cyclohexanedione	Cyclic ketones	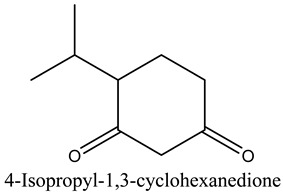	11.09	0.24
35	1-Ethylcyclohexene	Cycloalkenes	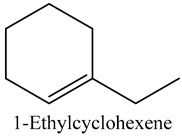	10.21	0.27
36	3-ethyl-2-methyl-1,3-Hexadiene	Dienes	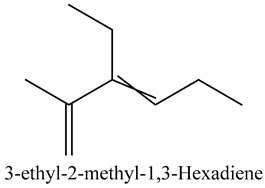	12.50	1.94
37	2,3-Octanedione (CAS)	Diketones	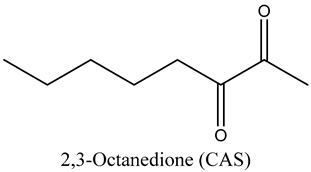	10.96	0.73
38	2-Amylfuran	Furans	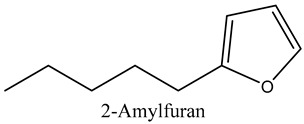	8.43	2.88
39	2-Phenoxyethanol	Glycol ethers	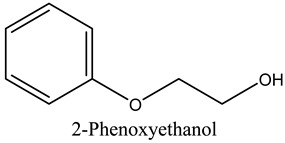	22.90	0.11
40	p-Ethylguaiacol	Guaiacols	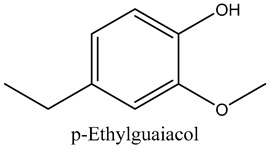	21.57	0.08
41	1,2-Dichlorobenzene	Halogenated benzenes	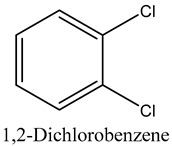	13.06	0.35
42	2-Nonanone	Ketones	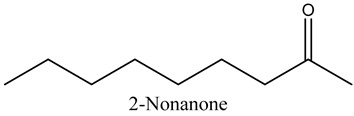	12.12	0.39
43	2-Octanone	Ketones	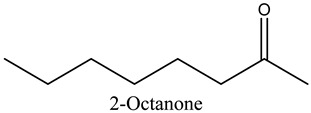	9.86	0.16
44	γ-Hexalactone	Lactone	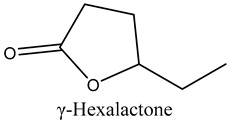	17.19	0.45
45	γ-Valerolactone	Lactone	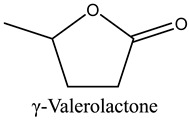	15.83	0.16
46	4-Pentylbutan-4-olide	Lactones	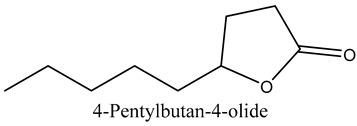	21.50	0.24
47	γ-Butyrolactone	Lactones	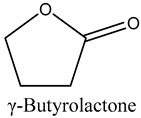	16.10	0.12
48	Heptanoic acid	Medium-chain fatty acid	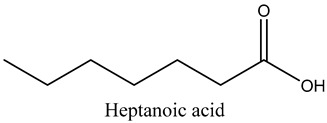	20.63	1.86
49	Linalool	Monoterpene alcohol	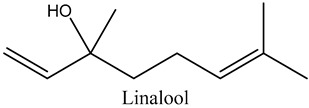	15.00	0.13
50	Limonene	Monoterpenes	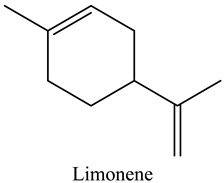	6.93	3.70
51	Guaiacol	Phenolic compounds	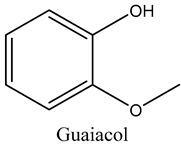	19.43	0.59
52	2,6-Dimethoxyphenol (Syringol)	Phenols	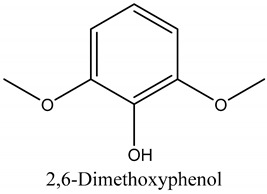	24.31	0.14
53	m-Cresol	Phenols	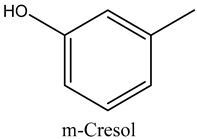	23.22	0.03
54	p-Cresol	Phenols	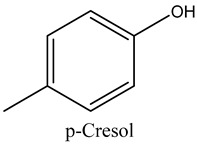	22.22	0.07
55	Phenol	Phenols	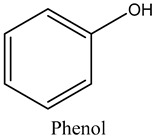	21.30	0.33
56	3-Ethylpyridine (β-Lutidine)	Pyridines	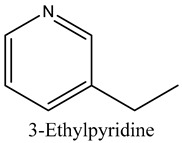	11.88	0.71
57	3-Vinylpyridine	Pyridines	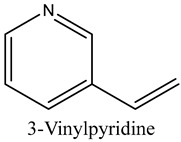	13.70	0.30
58	Hexadecanoic acid	Saturated fatty acids	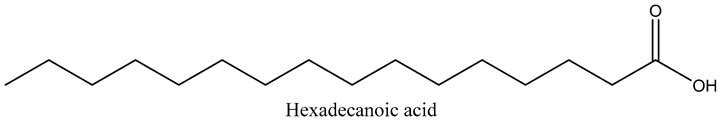	30.68	0.30
59	Hexanoic acid	Saturated fatty acids	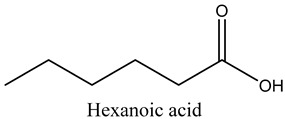	19.25	6.60
60	α-humulene	Sesquiterpene	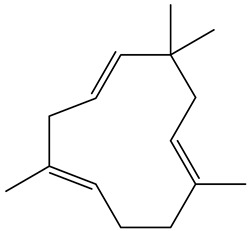	16.62	0.17
61	(+)-β-selinene	Sesquiterpenes	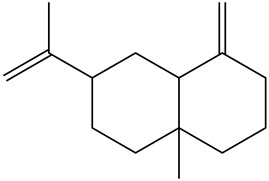	17.35	0.90
62	α-selinene	Sesquiterpenes	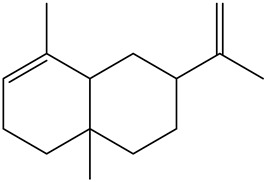	17.44	0.28
63	α-Ylangene	Sesquiterpenes	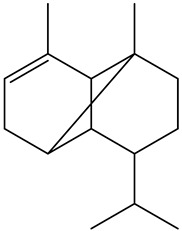	13.80	0.11
64	β-caryophyllene	Terpenes	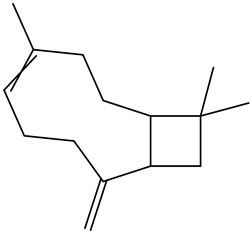	15.52	1.51
65	Styrene	Vinyl aromatic hydrocarbons	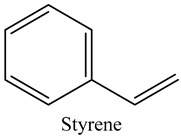	9.07	0.89

* Created using ChemDraw Version 20.1.1.

**Table 2:** *Sclerocarya birrea* kernel fatty acid methyl ester (FAME) constituents.

Component (Methyl Esters)	Abbreviation	Concentration (mg/g)
* α-Linolenic acid	C18:3n3	1.37
* Linoleic acid	C18:2 (cis)	55.54
Palmitic acid	C16	81.072
Palmitoleic acid	C16:1	0.994
Oleic acid	C18:1 (cis)	521.61
Lauric acid	C12	0.181
Heptadecanoic acid	C17	0.877
Stearic acid	C18	42.45
Arachidic acid	C20	3.46
cis-11-Eicosenoic acid	C20:1	1.37
Henicosanoic acid	C21	0.008
Behenic acid	C22	0.794
Tricosanoic acid	C23	0.082
Lignoceric acid	C24	0.863
Myristic acid	C14	0.354
Pentadecanoic acid	C15	0.076

* Essential fatty acids1.

## Data Availability

Data are contained within the article.
